# The Understanding and Exploration of Community Perception and Response Associated With Monkey Bites in a Rural District of Odisha, India: A Qualitative Study

**DOI:** 10.7759/cureus.77559

**Published:** 2025-01-16

**Authors:** Bhargavi Alajangi, Naisargika Jena, Abhisek Mishra, Swayam Pragyan Parida, Arvind K Singh, Subhashree Das

**Affiliations:** 1 Community Medicine and Family Medicine, All India Institute of Medical Sciences, Bhubaneswar, Bhubaneswar, IND

**Keywords:** animal ethics, animal human conflict, health system preparedness, monkey bite, one health, rabies

## Abstract

Background

Human-wildlife conflict is a global threat to sustainable development, food security, and conservation. Southeast Asia, including India, faces a major risk, with a very high number of animal bites occurring annually. While several studies have focused on dog bites, the aim of this study is to describe monkey bite cases and understand the associated factors to improve rabies prevention. This study explores patient perceptions and attitudes regarding the availability of services, alongside considerations of human and animal ethics with respect to monkey bites.

Methods

An exploratory qualitative study was conducted with bite victims using purposive sampling. In-depth interviews (IDIs) were employed to gather detailed information about the bites and associated factors until data saturation was achieved from four participants.

Results

Victims provided detailed accounts of the incident, their perceptions and attitudes regarding animal bites, and their ethical concerns. Content analysis of the transcripts revealed five themes: (i) bite as an event (nature of the bite, knowledge of the victims), (ii) post-event care (management at home, management at the hospital), (iii) health system (health system preference, health system preparedness), (iv) animal-human conflict (animal control measures, preventive measures), and (v) environmental and ethical issues.

Conclusion

There was a lack of knowledge regarding wound management and health service utilization among bite victims. The study highlights the need for awareness generation and educational sessions focused on environmental and animal ethics to improve health outcomes among the general public.

## Introduction

Human-wildlife conflict (HWC) refers to any interaction that negatively impacts human life, wildlife conservation, or the environment. Climate change and post-COVID-19 conditions have led to shifts in the habitat ranges of many species, amplifying competition for resources and altering established human-animal relationships [1,2]. According to the World Health Organization (WHO), 1.4 billion people in Southeast Asia are at heightened risk of rabies infection [3]. In India, approximately 18 human rabies cases are seen per one million population annually [4]. Each year, the National Rabies Control Programme in India reports around 130-210 rabies-related deaths in hospitals, while approximately seven million animal bites are reported annually as of 2019 [5]. Rabies, a fatal zoonotic illness, can be contracted through these bites and is responsible for 59,000 human deaths, 3.7 million disability-adjusted life years (DALYs), and economic losses of up to $8.6 billion globally each year [6]. In India, exposure to monkeys is a significant concern after dog bites, with the monkey population exceeding 50 million [7]. As per a report in 2016, more than 1,000 cases of monkey bites are reported daily in Indian cities [8].

Treatment for such exposures depends on immediate first aid, along with the administration of anti-rabies vaccine (ARV) and rabies immunoglobulin (RIG). Approximately 20,000 animal bites require post-exposure rabies prophylaxis; however, the availability of rabies immunoglobulin and vaccines varies widely between states. Immunoglobulins are used in only nine out of 27 (33.3%) government healthcare facilities and in two out of eight (25%) private facilities [9]. None of the studies indicated that patients regarded animal bites as emergencies, nor did they seek aid such as free ambulance services for transportation to the nearest health facility.

India, as a signatory to the Global Conference of Rabies Framework (2015), has adopted a “One Health” approach to achieve zero deaths from dog-mediated rabies by 2030, with Goa being the first state to achieve this goal [10,11]. The eastern state of Odisha, with 51,619 km² of forest land, faces a particularly high risk of HWC due to its 19 wildlife sanctuaries, one national park, and two tiger reserves [12]. This study aims to contribute to understanding the community's perception of animals holistically, along with their knowledge of monkey bite management. Even though there is a significant burden of monkey bites in India, the existing literature on it is subpar.

## Materials and methods

This was an exploratory qualitative study, utilizing in-depth interviews (IDIs), conducted at All India Institute of Medical Science (AIIMS), Bhubaneswar, Odisha, India, between June and July, 2023. This study was approved by the Institute Ethics Committee, AIIMs, Bhubaneswar (Letter no. T/IM NF/CM&FM/22/09). Respondents agreed to have their voices recorded for research purposes. The patient information sheet was read in the local language to explain the objective and purpose of the study. Written and verbal informed consent forms were signed, and verbal consent was also recorded in audio files.

Participants

Participants in the study were all monkey bite victims attending the immunization clinic at AIIMS, Bhubaneswar, from the village of Sarakantara, located 5 km from the study site. Purposive sampling and a consecutive method of respondent selection were employed. Participants were asked to recall the events of the bite and the actions they took afterward. Interviews were conducted face-to-face in a secluded corner of the immunization counseling room.

Study tool and interview

IDIs were conducted with a thematic analysis approach to understand all determinants as experienced by the study participants. The research team developed the interview guide following an extensive literature review to capture the perspectives of victims, with input from qualitative research experts. The guide included instructions for the interviewer and a semi-structured questionnaire (see Appendices) containing seven open-ended questions aimed at exploring the event of the monkey bite, participants' pre-existing beliefs, health system preferences/readiness, and animal-human ethics. Additional notes were taken post interview to capture any other relevant topics the respondents wished to discuss.

The trustworthiness of the research was ensured through multiple methods, including transferability, confirmability, and dependability. Transferability was achieved by providing detailed information about sampling, the study setting, participant demographics, and the semi-structured questions. Confirmability was established through a consensus-building approach among researchers in finalizing the codes; any discrepancies were resolved through discussions and meetings. Dependability was ensured by performing a thematic analysis according to Braun and Clarke's method. The interview guide was pre-tested, and the questions, probes, and instructions for the moderator were prepared following the Consolidated Criteria for Reporting Qualitative Research (COREQ) checklist.

The interviews were conducted by one researcher (VA), who received formal training and supervision from the principal researcher (AM) on conducting IDIs. The interviewer used the guide's leading questions and probes to elicit detailed descriptions from the participants. The average duration of the IDIs was 24 minutes (range: 20-29 minutes), and interviews continued until no new information was obtained. The interview guide is provided in the Appendices. 

Transcript preparation and analysis

Data were collected in the form of verbatim transcripts in the local language, which were later translated into English by the researcher (AM), who is fluent in both languages. Content analysis was conducted using an inductive coding approach. Open coding was initially performed by one researcher, and the codes were later merged to form domains and themes through a consensus-building approach (Braun and Clarke's method). Any disagreements between the researchers were resolved through discussion. All codes, domains (sub-themes), and themes were organized in Microsoft Excel sheets (Microsoft Corporation, Redmond, Washington, United States). Sociodemographic variables such as age, gender, place of residence, educational qualification, occupation, and contact information for each participant were recorded. The identified themes included: the event, post-event care, the health system, human-animal conflict, environment, and ethical issues.

## Results

Participant characteristics

Four female participants, aged 9-70 years, from the village of Sarakantara, were interviewed in our study setting. Two of the participants were school-going children, one was a homemaker, and one was an Anganwadi worker (frontline worker). All participants belonged to a lower socioeconomic class. Table 1 summarizes additional participant variables.

**Table 1:** Sociodemographic characteristics of study participants

Subject Characteristics		Participant 1	Participant 2	Participant 3	Participant 4
	Gender	Female	Female	Female	Female
	Age (in completed years)	9	70	12	63
	Education (Last education achieved)	Class 5	Class 10	Class 7	Class 5
	Occupation	Student	Home maker	Student	Anganwadi worker
	Average Family income	10000	10000	12000	10000
Bite characteristics	Site of the bite	Left ankle	Right ankle and forehead	Back and over shoulders	the left hand near the wrist
	Time of the bite	3 PM	3 PM	2:30 PM	10:15 AM
	First point of health care facility	AIIMS	AIIMS	Subcentre	AIIMS
	Time since the first point of contact with the Hospital/Rabies clinic	Within 2 hrs	Within 2 hrs	Within 1 hr	Within 1 hr
	Wound wash done for 10-15 minutes	No	No	No	No
	Wound area washed with soap	No	No	Yes	No
	Wound area applied Dettol or local antiseptic	No	No	No	Applied some natural medication

Three out of four bites occurred in the afternoon, all categorized as Category III (most severe) and were non-provoked. The health system preference was government hospitals, and none of the participants properly washed their wounds. Timely presentation to a hospital or treating physician is the most important factor in animal bite cases. All participants presented to healthcare facilities at different time intervals.

Theme 1: bite as an event

All victims described their monkey bite incidents based on their prior knowledge and the factors they recalled.

Subtheme 1: Nature of the Bite

This subtheme highlights that all bites were severe, Category III, non-provoked, and mainly occurred on the ankle and back. The bites happened in the afternoon when students were returning from school and the homemaker was outside for work.

Participant #1: *“It didn’t bite, it scratched. I was going on a bicycle, like always returning from school and suddenly a monkey came onto me from behind and scratched me over my arms it started bleeding and I got scared.”*

Subtheme 2: Response to the Bite

In this subtheme, participants shared their understanding of the necessary actions, healthcare facility preferences, and their knowledge of ARV and RIG. Although participants’ knowledge of wound management was limited, all reported washing the wound.

Participant #3: “*We didn’t know what to do in that case so, we applied some locally available herb and then washed the wound with soap and water for 2 minutes.”*

Participant #4: *“Similar thing happened in the morning, our neighbors were bitten by monkey, they only suggested us to visit AIIMS to get free treatment”*

Theme 2: post-event care

This theme examines how participants managed the bites at home, at the first point of contact, and in the hospital. In the subtheme "management at the first point of care," it was observed that participants always started care at home. Contrary to our expectations, they washed their wounds and applied locally available remedies, but the thoroughness of the washing was inadequate.

Participant #2:

 “We didn’t know what to do, an hour later we washed the wound at home and then we went to the hospital.”

In one case, locally available leaves were applied to the wound, which is not recommended. However, all participants eventually sought medical care at a nearby government hospital.

 Participant #3:

 “I applied ‘Kalara patra’ and then washed the wound with soap and water for 2 minutes and came to the hospital”

In the second sub-theme ‘management at hospital’, they have described the procedures done at the government health facility.

Participant #2:

They asked about the wound-washing technique, how long we used soap and water, and whether we applied pressure while washing. They also asked if we had taken a tetanus injection?”

We found that counseling on proper wound-washing techniques such as keeping the wound under running water for 10-15 minutes, using soap, and applying antiseptic was provided by the nursing staff. All participants, having suffered Category III bites, received ARV and Equine Rabies Immunoglobulin (ERIG), and were counseled on follow-up doses. Participants were also told they would receive a certificate upon completing the ARV schedule.

Participant #4:

 “They advised us to come for further doses and told they would provide a certificate after the completion of all doses”

Theme 3: health system factors

Participants shared their experiences with the health system, including issues and challenges. All participants preferred government hospitals, whether a nearby sub-center or AIIMS, a tertiary care hospital. Although government services were preferred due to affordability, accessibility, and availability, there was no involvement of Accredited Social Health Activist (ASHA) or Anganwadi workers in their cases

Participant #1:

 “We went to the nearby government hospital, it is accessible, convenient and nearer to our home so we chose to go to the government hospital.”

Participants have opined about the health system preparedness regarding the readiness of the attended health facility. All of them mentioned the healthcare workers' knowledge and attitude regarding their wound management. They felt there was a lack of availability of services at the primary care level nearest to their village or community.

Participant #4:

“Health-care providers had knowledge to manage the case but injections were not available in that hospital, so they referred us to AIIMS hospital.”

Theme 4: animal-human conflict:

Participants described their experiences with animal-human conflict and possible solutions, including rescue and relocation efforts. The subtheme "Animal control measures" highlighted societal attitudes toward wild animals. Though people were scared of the monkeys, no one attempted to kill them, possibly due to religious beliefs.

Participant #2:

“Exactly we didn’t know what to be done, people around us were scared, by this time the monkey had already bitten around 8 people from our village in over 2 days.”

As said by another respondent, the knowledge about animal rescue and repositioning in the nearest animal shelters was lacking among the villagers. They were unaware of any specific organization or individual addressing this issue and believed it was the responsibility of their community leader to manage such incidents, which could occur at any time.

Participant #3:

“Other day, animal rescue team came and caught the monkey without harming him. Our sarpanch knew who to call what to do so he called the rescue team.”

On asked about ‘preventive measures to reduce animal-human conflicts’, a gap in knowledge was observed among respondents regarding the control measures for HWCs. One subject highlighted people need to be educated through information, education, and communication (IEC) campaigns on how to deal with wild animals. They emphasized the use of prominent signs in the local language to ensure public safety in areas with significant wildlife presence.

Participant #1:

“I think public awareness matters a lot, people should not feed or tease animals at all. Posters/sign boards should be placed in the places where there more animal bite prevalence”

Theme 5: environment and ethical issues

Several respondents expressed ethical concerns related to animal-human conflicts. They frequently encountered monkeys, especially during harvest season, and were worried about crop damage and food wastage. Although they understood that it was wrong to harm animals, they were unsure about how to handle these conflicts.

Participant #4:

 “A troop of monkeys come to raid our food supplies, and some villagers and children beat the monkeys when they try to take it. We know it's wrong, but that's how we manage to stop them.”

The themes are described in Table 2.

**Table 2:** Description of themes, sub-themes, and categories IEC: information, education, and communication

Themes/Categories	Subcategories	Codes
Bite as an Event	Nature of the Bite	Not Provoked, Wild Animal, Scratch and Bleeding, Vulnerable population, Time of Bite
	Knowledge of the Victims	Inadequate knowledge, Knowledge from Neighbors, Regular phenomena, Free service at the hospital
Post-event care	Management at the Home or Locality	Washed the Wound, Application of Natural Medication, Self-Referral
	Management at hospital	Appropriateness and Readiness, Follow up, Unavailability at first point of contact, Counselling, Certificate
Health system	Health system preference	Government Hospital, Easy accessibility, Free Treatment, Convenient
	Health system preparedness	Adequate knowledge at Tertiary care, Good behavior and practice, Follow up.
Animal human conflict	Animal control measures	Lack of information, Local Authorities, Animal Rescue Team
	Preventive measures	Regular Public Awareness, IEC Display, Animal feeding practices, Vigilance Regarding Monkey Menace
Environment and Ethical Issues		Mobility from local forest, Cruelty towards Animal, Non-Ethical Practices, Attacking Food Sources, Animal Habitat

## Discussion

In this study, we observed that all exposures were severe, consistent with findings from a study in southern Odisha where the majority of bites involved dogs (76%) and were severe [13]. The bite locations, frequently on the lower and upper limbs, also aligned with the findings of our study. In our study, all victims were female, either students or elderly, indicating greater vulnerability at extreme ages. Notably, none of the victims with severe bites considered their situation an emergency, as none sought ambulance services, even when available for free.

A study in urban South India by Sivagurunathan et al. on animal bites and rabies found unsatisfactory knowledge, attitudes, and practices (KAP) scores. Females were more at risk than males due to inadequate knowledge about animal bites, rabies, first aid, ARV (anti-rabies vaccine), and tetanus immunization following animal bites [14]. Similarly, our study also observed unsatisfactory knowledge regarding bite management. None of the participants consulted frontline health workers, even though they considered the bites a medical emergency. In another study from Thailand, only 54.5% of school students recognized the severity of rabies, highlighting a significant knowledge gap about the disease [15].

In the current study, none of the victims properly washed their wounds. A study from Kerala found that only 19% of the population had poor knowledge of first aid, and none of them had good knowledge regarding proper wound washing [16]. Even though one of our participants was an Anganwadi worker, a clear knowledge gap was observed. One participant used "Kalara Patra," a commonly available herb. Despite claims by traditional healers of 98% success in preventing rabies deaths, no controlled trials or community surveys support its efficacy. All victims preferred government hospitals, possibly due to their rural setting and awareness of the free services available. A study from Rajasthan reported that female victims were less likely to seek treatment in comparison to male [17].

A study on knowledge and practices about animal bite management among government doctors posted at primary health-care settings of district Patiala in Punjab found that only 60% of doctors were aware of the correct post-exposure prophylaxis (PEP) schedule and less than half of the respondents knew the appropriate methods for wound washing and first-aid protocols [18]. A study done on knowledge of animal bite management and rabies immunization among interns of a government medical college in Kolkata found that around 70% of interns correctly identified rabies as a fatal disease; however, only 40% were aware of the WHO-recommended intradermal rabies vaccine schedule [19]. Misconceptions about wound washing and first aid were common, with less than 50% knowing the importance of using soap and water immediately after a bite. In the current study, the tertiary care facility was reported by the victims to be well-prepared to manage bite victims with knowledgeable staff.

Several studies point to inadequate knowledge among participants regarding animal-human conflicts and necessary measures. Like others, our study too did not observe healthcare workers' involvement in this aspect. The community showed a significant lack of knowledge about immediate responses, such as engaging an animal rescue team. Community leaders play a crucial role in deciding how to handle encounters with wild animals [20,21]. According to Madden and McQuinn, HWCs often revolve around resource and security issues such as damage to crops, cattle, fisheries, game, property, or facilities [22]. This conflict is categorized as Level 2 due to recurring issues with no adequate resolution.

Ethics in HWC involves a universal, interdisciplinary dialogue that uses logic and evidence to promote the welfare of humans, animals, and the environment. In the current study, there was no killing or capturing of the wild animal. Instead, the local leader called the rescue team, and the animal was safely relocated. The National Guidelines for Rabies Prophylaxis of the National Rabies Control Programme set by the Government of India focuses on ensuring peaceful coexistence and the ethical treatment of animals [5]. There is scant literature that emphasizes the importance of considering rural and local perspectives to promote coexistence [23,24]. Habitat management, another key strategy for preventing HWCs, was not mentioned by any participant in our study. According to Reid and Miller, historically, buffer zones were defined as a collar of land managed to filter out inappropriate influences from surrounding activities [25]. However, they say the present day requires the management of these buffer or transition zones to be included in the management and financial planning of protected areas. A study from Thailand highlighted the establishment of buffer zones, which significantly reduced the impact of HWCs [26].

Limitations of the study

Animal bites, particularly those from monkeys, are understudied in many regions. This qualitative exploratory study focuses on the perceptions of a small sample of victims, which limits the generalizability of the findings to the broader population. Additionally, this study did not explore healthcare workers' perspectives or assess the readiness of healthcare facilities to manage animal bites. Furthermore, key stakeholders involved in animal rescue, relocation, and resettlement were not interviewed, which limits insight into the broader system of animal-human conflict management.

## Conclusions

Our analysis provides valuable insights into community perceptions of animal bite incidents, as well as their views on animal and human ethics. The bite victims demonstrated a preference for seeking care at government health facilities, but they did not perceive the bites as medical emergencies. To address this, capacity-building initiatives for frontline workers should be implemented in areas prone to HWCs, with a focus on educating the general public, particularly vulnerable groups like children, adolescents, and the elderly, about proper wound management.

It is crucial to establish accountability within the current healthcare system to ensure quality and comprehensive bite management at facilities closer to the community, ideally at the primary healthcare level. Additionally, public awareness about preventing HWCs should be increased through IEC programs or behavior change communication (BCC) activities, involving key stakeholders. Practical ethics are vital for promoting peaceful coexistence between humans and animals in conflict-prone areas, in alignment with the principles of the "One Health" approach.

## Appendices

Moderator guide

Greetings! I appreciate your interest in participating in this activity. We are very interested to hear your valuable opinion on the factors associated with animal bites (Monkey bites) for our study. We would like to record the interview so that we can make sure to capture the thoughts, opinions, and ideas we hear from you. No names will be attached and the tapes will be destroyed as soon as they are transcribed. You may refuse to answer any question or withdraw from the study at any time. We understand how important it is that this information is kept private and confidential. Please sign the sheet that will be handed out to you to show you agree to participate in this interview.

Total duration: 8-10 minutes

Venue: Seminar room, Immunisation Clinic, AIIMS Bhubaneswar

In-depth interview of the understanding and exploration of factors associated with animal bites in the rural community of Sarakantara village in Khordha district

Questions and Probes

1.     Tell us about yourself

•                  Life, occupation, and employment.

•                  Any worry or anxiety

2.     Describe in detail the animal bite that you faced.

•                  Time, place, and site of the bite

•                  How did the bite occur, Nature of the bite?

3.     Tell us your knowledge about the post-bite behavior and events

•                  Wound management, cleaning, and debridement

•                  TT Vaccine, ARV, and RIGs

4.     Health system preference by you, Discuss in detail

•                  Govt/Private/Clinic

•                  Any self-medication

5.     Elaborately explain the health system preparedness that you have witnessed

•                  Knowledge of the personnel

•                  Wound care practices, Management including vaccination and follow-up

•                  Availability of the logistics

•                  Counselling

6.     Tell us in detail about the Ethical issues that could arise due to Animal-Human conflict

•                  Human aspect

•                  Animal Aspect

7.     Any Animal control measures that are in your knowledge are being done

•                  Animal control activities

•                  Involvement in other sectors
